# Motion control and positioning system of multi-sensor tunnel defect inspection robot: from methodology to application

**DOI:** 10.1038/s41598-023-27402-z

**Published:** 2023-01-05

**Authors:** Ke-Qiang Liu, Shi-Sheng Zhong, Kun Zhao, Yang Song

**Affiliations:** 1 School of Mechatronics Engineering, Harbin Institute of Technology, Harbin, China; 2 CRRC Qingdao Sifang Vehicle Research Institute Co., Ltd, Qingdao, China

**Keywords:** Civil engineering, Mechanical engineering

## Abstract

As the mileage of subway is increasing rapidly, there is an urgent need for automatic subway tunnel inspection equipment to ensure the efficiency and frequency of daily tunnel inspection. The subway tunnel environment is complex, it cannot receive GPS and other satellite signals, a variety of positioning sensors cannot be used. Besides, there are random interference, wheel and rail idling and creep. All the above results in poor performance of conventional speed tracking and positioning methods. In this paper, a multi-sensor motion control system is proposed for the subway tunnel inspection robot. At the same time, a trapezoidal speed planning and a speed tracking algorithm based on MPC (Model Predictive Control) are proposed, which simplify longitudinal dynamics model to overcome the complex and variable nonlinear problems in the operation of the maintenance robot. The optimal function of speed, acceleration and jerk constraint is designed to make the tunnel inspection robot achieve efficient and stable speed control in the subway tunnel environment. In this paper, the "INS (inertial navigation system) + Odometer" positioning method is proposed. The difference between the displacement measured by the inertial navigation system and the displacement calculated by the odometer is taken as the measurement value, which reduces the dimension of the conventional algorithm. The closed-loop Kalman filter is used to establish the combined positioning model, and the system error can be corrected in real time with higher accuracy. The algorithms were verified on the test line. The displacement target was set to be 1 km and the limit speed was 60 km/h. The overshooting error of the speed tracking algorithm based on trapezoidal velocity planning and MPC was 0.89%, and the stability error was 0.32%. It improved the accuracy and stability of the speed following, and was much better than the PID speed tracking algorithm. At the speed of 40 km/h, the maximum positioning error of the robot within 2 km is 0.15%, and the average error is 0.08%. It is verified that the multi-sensor fusion positioning algorithm has significantly improved the accuracy compared with the single-odometer positioning algorithm, and can effectively make up for the position error caused by wheel-rail creep and sensor error.

## Introduction

With the advancement of urbanization around the world, the urban population is increasing, and the increasingly prominent problem of urban congestion promotes the rapid development of urban rail transit industry. Under the background of the climax of subway construction, with more and more subway lines put into operation, many structure problems are gradually exposed in the subway tunnels that have been opened to traffic, such as cracks, deformation, leakage, etc.^1^ The daily allowable detection window of subway tunnel is 2–2.5 h, and the maintenance workload is heavy.

How to complete a large number of detection work in a limited time during tunnel operation will be the focus in the future. In view of the problems of small number of measuring points, slow detection speed and large amount of manual work existing in the traditional tunnel detection technology, there is an urgent need for an efficient and economical detection technology and equipment, which can quickly complete the measurement, ensure the measurement accuracy, be economical and durable, and maximize the automation.

At present, there are many researches on disease recognition of detection equipment in the world^2–5^. But the motion control is not intelligent, mainly manual driving or operation, and cannot adapt to the complex tunnel environment. The detection data is processed manually, the positioning efficiency is slow.

## Discussions

At present, there are three main speed tracking algorithms for autonomous rail vehicles.

1. Classical control algorithm based on PID and improved algorithm based on PID: It performs well in general working conditions, but the performance deteriorates under traction and brake switching. At the same time, as the vehicle system often has strong nonlinear and too many constraints, how to determine the optimal PID parameters becomes a challenge. Researchers have tried the PID control algorithm based on particle swarm optimization algorithm^6^, fuzzy prediction^7^ and other parameters self-tuning.

2. Algorithm based on intelligent control: Artificial intelligence algorithms such as neural network^8^, iterative learning^9^ and expert system^10,11^ transform driving knowledge and experience into a series of domain rules through original data to simulate the driving strategy of people with rich experience, which can overcome the dependence on accurate mathematical models. But it is highly dependent on artificial design. It often requires a large amount of test data and parameter calibration to make rule base^12^.

3. Adaptive algorithm: Researchers use adaptive control algorithm^13^, back-step method^14^, synovial control^15^, etc., to overcome the shortcomings of PID algorithm and intelligent control algorithm, and achieve some results. In recent years, model predictive control (MPC) and its evolution algorithm have been used in motor control^16^, path tracking^17–19^, microgrid energy management^20^, energy-efficient buildings^21^, biological fermentation^22^ and other applications have good results. MPC algorithm has the advantages of simple model requirements, small amount of computation, fast response speed and smaller overshoot. Inspired by these applications, combined with the longitudinal motion model of tunnel inspection robot and a variety of constraints, the improved MPC algorithm is adopted for speed tracking.

The inspection robot needs to obtain accurate positioning in real time for speed tracking and tunnel disease location information. At present, rail vehicles cannot be accurately positioned through a single sensor such as a rotating shaft speed sensor, but also need to manually mark mileage markers or lay transponders in the tunnel. The cost is very high, and the intermediate process is not accurate positioning. Multiple methods have been studied in robot positioning. Literature^23,24^ proposes a new denoised stereo visual odometry VO/INS/GPS integration system for autonomous navigation based on tightly coupled fusion, which is used to estimate vehicle position in GPS rejection or low texture environment. Literature^25^ designed the Kalman filter and GCC1 fusion method based on the optical flow sensor and the inertial sensor of UAV to carry out the localization algorithm of the underground cable tunnel detection. Literature^26^ designed a positioning system by capturing high-resolution images of the inner surface of the tunnel to create detailed 3D models. Reference^27^ developed VILENS (Visual Inertial Lidar Legged Navigation System), an odometry system for legged robots based on factor graphs. Literature^28^ reviewed the positioning of underground coal equipment by using Kalman filter combined with velocity observation mode.

Literature^29^ summarized the experience and lessons of robot research in tunnel environment in nearly 10 years, and pointed out that due to the complex environment of iron tunnel, satellite signals such as GPS and other signals could not be received, various positioning sensors could not be used, there were many uncertain factors, excessive structural attachments, random interference, wheel and rail idling and coasting. May result in the failure of algorithms such as tachometer localization, scanning-matching, LiDAR based SLAM, or visual SLAM. Literature^30^ conducted in-depth analysis and research on the optimal information fusion estimation algorithm, established the algorithm model of speed measurement and positioning system, and applied the classical information fusion algorithm to the speed measurement and positioning of trains in urban rail transit. In literature^31^, based on the combination positioning of pulse sensor, Doppler and transponder, and combined with the location fingerprint positioning principle of WKNN, a subway train combination positioning system based on CBTC was designed. However, the research of positioning algorithm focuses on the simulation stage, and the calculation amount is too large, which cannot completely overcome the complex underground tunnel environmental impact, and the construction cost is too high, so the actual use effect cannot reach the expected value.

In order to improve the intelligent operation of detection equipment, this paper takes the intelligent operation of subway tunnel inspection robot as the entry point to study its speed control and positioning algorithm. The main research contents and innovations are as follows:

1. Considering the short gap period for subway maintenance, in order to save vehicle running time and improve inspection efficiency as far as possible under the premise of safety, the trapezoidal speed planning algorithm was designed based on the running route and the maximum speed limit of different routes. Different speeds were matched in inspection section and cruise section, and the braking distance for obstacle detection was reserved.
2. After the velocity planning curve is obtained, in order to realize the automatic and accurate control of the robot's velocity, the MPC speed tracking algorithm is adopted, and the simplified longitudinal kinematics model is used to overcome the complex and changeable nonlinear problems in the operation process of the maintenance robot. Besides, the constrained optimal function of velocity, acceleration and jerk is designed. The algorithm will reduce the vibration of the maintenance robot and realize the speed tracking of the robot quickly and accurately.
3. Without adding external positioning landmarks, the positioning algorithm of "INS + Odometer" fusion is adopted. The difference between the displacement measured by inertial navigation system and the displacement calculated by odometer is taken as the measurement value. The closed-loop Kalman filter is applied to establish the combined positioning model, which has fewer matrix dimensions than the traditional velocity observation mode and can correct the sensor errors in real time. Moreover, it can provide accurate position information for robot speed control and disease monitoring.

### Ethics approval

I would like to declare on behalf of my co-authors that the work described was original research that has not been published previously, and not under consideration for publication elsewhere, in whole or in part. All the authors listed have approved the manuscript that is enclosed. Research did not involve Human Participants and/or Animals.

### Consent to participate

All authors agreed to participate in the project.

## System scheme design

### System structure

**Figure 1:**
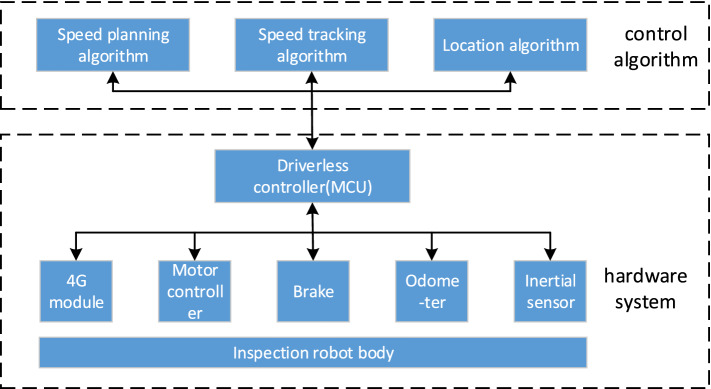
Structural diagram of motion control and positioning system of tunnel inspection robot.

**Figure 2:**
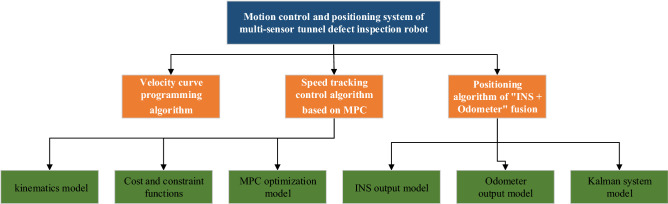
The structure of the algorithm.

The motion control and positioning system of tunnel inspection robot mainly includes two parts: hardware system and control algorithm, shown in Fig. 1. The hardware system includes 4G module for network communication, motor controller for motor torque control, brake for emergency braking, odometer and inertial sensor for positioning; The control algorithm mainly includes speed planning algorithm, speed tracking algorithm and fusion of inertial navigation and odometer positioning algorithm. The structure of the algorithm is shown in Fig. 2.

### Velocity curve programming algorithm

**Figure 3:**
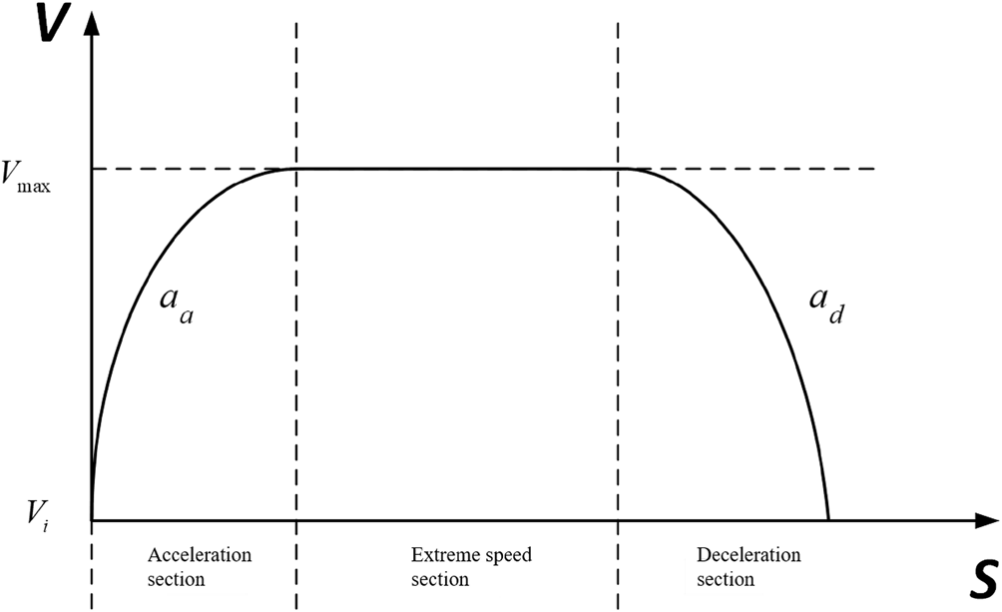
Basic principle of trapezoidal speed planning.

The basic principle of trapezoidal speed planning^32^ is shown in Fig. 3. The local path planning length (or distance from the obstacle length) of the inspection robot is S. The inspection robot starts at the initial speed $V_{i}$, passes through the acceleration section, extreme speed section and deceleration section, and finally stops at the point S at zero speed, which is the maximum speed limited $V_{\max }$ by the inspection robot. In order to avoid excessive acceleration and wheel slip, the maximum acceleration is limited to $a_{a}$. In the deceleration section, the maximum acceleration is limited to $a_{d}$. The motion process of inspection robot can be extended by this planning algorithm.

At this time, the calculation formula of the distance required for acceleration section, deceleration section and extreme speed section is:

1 $$\left\{ \begin{gathered} S_{a} = \left\{ {\begin{array}{*{20}c} {\tfrac{{V_{\max }^{2} - V_{i}^{2} }}{{2a_{a} }},\begin{array}{*{20}c} {} & {V_{i} < V_{\max } } \\ \end{array} } \\ {0,\begin{array}{*{20}c} {} & {} \\ \end{array} \begin{array}{*{20}c} {} & {V_{i} \ge V_{\max } } \\ \end{array} } \\ \end{array} } \right. \hfill \\ S_{d} = \tfrac{{ - V_{i}^{2} }}{{2a_{d} }} + \alpha V_{i} \hfill \\ S_{m} = S - S_{a} - S_{d} \hfill \\ \end{gathered} \right.$$

where $\alpha V_{i}$ represents the traveling distance between the upper computer sending the braking instruction and the actuator executing the braking action. $S_{a}$ is the acceleration displacement. $S_{d}$ represents the displacement of deceleration section. $S_{m}$ indicates rapid short displacement.

The result of trapezoidal speed planning is the discrete expected value of speed, so it is necessary to segment the planned distance. Based on the step calculation method of initial vehicle speed, if its execution period is t, the driving distance within a cycle is $tV_{i}$, then the formula for step calculation is as follows:

2 $$l_{step} = tV_{i}$$

**Figure 4:**
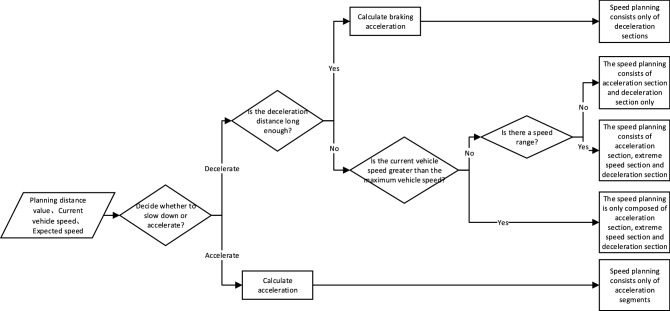
Trapezoidal speed planning process.

According to the difference of current speed and planned distance length, the speed planning results can be divided into the several situations in the Fig. 4.

*Case 1*. If $V_{i} > V_{\max }$ and $S_{d} \ge S$ (or $V_{i} \le V_{\max }$ and $S_{d} > S$), the deceleration distance is insufficient, and emergency braking measures should be taken. According to the length of the planned path, the emergency braking speed reduction can be calculated to realize the vehicle deceleration and stopping action.

When deceleration distance is insufficient, there is only deceleration section in the speed planning result, and the deceleration value of emergency braking should be calculated according to the speed and planning distance. The calculation formula is as follows:

3 $$a_{e\_d} = \frac{{ - V_{i}^{2} }}{{2(S - \alpha V_{i} )}}$$

In the above formula, $a_{e\_d}$ represents the desired deceleration required for emergency braking.

At this time, the calculation formula of speed planning is:

4 $$V_{ei} = \left\{ \begin{gathered} \sqrt {V_{e(i - 1)}^{2} + 2a_{e\_d} l_{step} } \begin{array}{*{20}c} , & {S_{i} \le L_{1} } \\ \end{array} \hfill \\ 0\begin{array}{*{20}c} {\begin{array}{*{20}c} {\begin{array}{*{20}c} {\begin{array}{*{20}c} {\begin{array}{*{20}c} {\begin{array}{*{20}c} , & {} \\ \end{array} } & {} \\ \end{array} } & {} \\ \end{array} } & {} \\ \end{array} } & {} \\ \end{array} } & {L_{1} < S_{i} \le S} \\ \end{array} \hfill \\ \end{gathered} \right.$$

where $V_{ei}$ represents the planning speed of the ith point on the planning path. $S_{i}$ represents the distance from the start to zero deceleration. $L_{1} = S - \alpha V_{i}$ represents the distance from the start to zero deceleration.

*Case 2*. If $V_{i} > V_{\max }$ and $S_{d} < S$, the initial speed is higher than the maximum limit speed and the planned path is long enough, the speed planning will first slow down the vehicle to the maximum limit speed, drive at the limit speed for a distance, and then slow down to stop.

The distance travelled at each stage can be calculated:

5 $$\left\{ \begin{gathered} L_{1} = \frac{{V_{\max }^{2} - V_{i}^{2} }}{{2a_{d} }} \hfill \\ L_{2} = S - L_{3} \hfill \\ L_{3} = S - \alpha V_{i} \hfill \\ \end{gathered} \right.$$

where $L_{1}$ represents the distance required to decelerate from the current speed to the maximum speed limit, and $L_{3}$ represents the distance required to decelerate from the maximum speed limit to the desired speed zero.

The calculation formula of speed planning is:

6 $$V_{ei} = \left\{ {\begin{array}{*{20}l} {\sqrt {V_{e(i - 1)}^{2} + 2a_{d} (S_{i} - S_{i - 1} )} ,} \hfill & {\quad S_{i} \le L_{1} } \hfill \\ {V_{\max } ,} \hfill & {\quad L_{1} < S_{i} \le L_{2} } \hfill \\ {\sqrt {V_{e(i - 1)}^{2} + 2a_{d} (S_{i} - S_{i - 1} )} ,} \hfill & {\quad L_{2} < S_{i} \le L_{3} } \hfill \\ {0,} \hfill & {\quad L_{3} < S_{i} \le S} \hfill \\ \end{array} } \right.$$

*Case 3*. If $V_{i} < V_{\max }$ and $S_{d} < S$, it is necessary to determine whether there is a maximum speed segment in the speed planning result. If there is a maximum speed segment, the vehicle will first accelerate to the limit speed, drive at a constant speed for a distance, and then slow down to stop.

Calculate the distance traveled at each stage:

7 $$\left\{ \begin{gathered} L_{1} = S_{a} \hfill \\ L_{2} = S - L_{3} \hfill \\ L_{3} = S - \alpha V_{i} \hfill \\ \end{gathered} \right.$$

where, $L_{1}$ represents the distance required to accelerate from the current speed to the maximum speed limit, and $L_{3}$ represents the distance required to decelerate from the maximum speed limit to the desired deceleration speed to zero speed.

The calculation formula of speed planning is:

8 $$V_{ei} = \left\{ {\begin{array}{*{20}l} {\sqrt {V_{e(i - 1)}^{2} + 2a_{a} (S_{i} - S_{i - 1} )} ,} \hfill & {\quad S_{i} \le L_{1} } \hfill \\ {V_{\max } ,} \hfill & {\quad L_{1} < S_{i} \le L_{2} } \hfill \\ {\sqrt {V_{e(i - 1)}^{2} + 2a_{d} (S_{i} - S_{i - 1} )} ,} \hfill & {\quad L_{2} < S_{i} \le L_{3} } \hfill \\ {0,} \hfill & {\quad L_{3} < S_{i} \le S} \hfill \\ \end{array} } \right.$$

*Case 4*. If $V_{i} < V_{\max }$ and $S_{d} < S$, and there is no top speed segment, the vehicle first accelerates to the maximum intermediate speed, and directly decelerates and stops after the acceleration.

9 $$V_{c} = \sqrt {\frac{{2a_{a} a_{d} (S - \alpha V_{i} ) + a_{d} V_{i}^{2} }}{{a_{d} - a_{a} }}}$$

The distance travelled at each stage can be calculated:

10 $$\left\{ \begin{gathered} L_{1} = \frac{{V_{c}^{2} - V_{i}^{2} }}{{2a_{a} }} \hfill \\ L_{2} = S - \alpha V_{i} \hfill \\ \end{gathered} \right.$$

where $L_{1}$ represents the distance required to accelerate from the current speed to the highest intermediate speed, and $L_{2}$ represents the distance required to decelerate from the starting position to zero speed.

11 $$V_{ei} = \left\{ {\begin{array}{*{20}l} {\sqrt {V_{e(i - 1)}^{2} + 2a_{a} (S_{i} - S_{i - 1} )} ,} \hfill & {\quad S_{i} \le L_{1} } \hfill \\ {\sqrt {V_{e(i - 1)}^{2} + 2a_{d} (S_{i} - S_{i - 1} )} ,} \hfill & {\quad L_{1} < S_{i} \le L_{2} } \hfill \\ {0,} \hfill & {\quad L_{2} < S_{i} \le S} \hfill \\ \end{array} } \right.$$

*Case 5*. If $V_{i} = V_{\max }$ and $S_{d} < S$, when the vehicle speed is equal to the maximum speed limit and the planned distance is long enough, the vehicle first travels at the current speed for a distance, and then decelerates and stops.

So the acceleration $S_{a}$ is equal to zero, which is the same thing as case 3.

### Speed tracking control algorithm based on MPC

#### Kinematics model

Firstly, the kinematics model of the inspection robot is established. Because there are various nonlinearities of the motor, the creep of the wheel and the aerodynamic resistance, the kinematics model of the inspection robot is a complex nonlinear structure. In order to overcome this problem, considering that there is always time delay in the actual longitudinal dynamic state due to the slow response of the vehicle drive system, a first-order lag is introduced to approximately reflect the longitudinal motion characteristics of the vehicle^33^. Expected acceleration $a_{des}$ can be found in terms of the expected velocity $v_{ref}$ and the current velocity $v$. The relationship between the expected acceleration $a_{des}$ and the actual acceleration $a$ of the inspection robot can be expressed as by the first-order delay system:

12 $$a = \frac{K}{{\tau_{d} s + 1}}a_{des}$$

where $K = 1$ is the system gain, $\tau_{d}$ is the time constant.

The state equation of continuous system can be expressed as:

13 $$\begin{aligned} \dot{\user2{x}} & = \user2{\Phi x} +{\varvec{\varPi}}u \\{\varvec{\varPhi}}& = \left( {\begin{array}{*{20}c} 0 & 1 \\ 0 & { - {1 \mathord{\left/ {\vphantom {1 {\tau_{d} }}} \right. \kern-0pt} {\tau_{d} }}} \\ \end{array} } \right)\begin{array}{*{20}c} {} \\ \end{array}{\varvec{\varPi}}= \left( {\begin{array}{*{20}c} 0 \\ {{K \mathord{\left/ {\vphantom {K {\tau_{d} }}} \right. \kern-0pt} {\tau_{d} }}} \\ \end{array} } \right) \\ {\varvec{x}} & = \left( {\begin{array}{*{20}c} v & a \\ \end{array} } \right)^{T} \quad u = a_{des} \\ \end{aligned}$$

where ${\varvec{x}} \in {\varvec{R}}^{2}$ is the system state vector; $u \in {\varvec{R}}$ is the system control input.

Using the Forward Euler method, the discrete state equation of the system is obtained:

14 $$\begin{aligned} & {\varvec{x}}(k + 1) = {\varvec{Ax}}(k) + {\varvec{Bu}}(k) \\ & {\varvec{A}} = \left( {\begin{array}{*{20}c} 1 & T \\ 0 & {1 - {T \mathord{\left/ {\vphantom {T {\tau_{d} }}} \right. \kern-0pt} {\tau_{d} }}} \\ \end{array} } \right)\begin{array}{*{20}c} {} \\ \end{array} {\varvec{B}} = \left( {\begin{array}{*{20}c} 0 \\ {{{KT} \mathord{\left/ {\vphantom {{KT} {\tau_{d} }}} \right. \kern-0pt} {\tau_{d} }}} \\ \end{array} } \right) \\ \end{aligned}$$

where $k$ is the current sampling time; $k + 1$ is the next sampling time; $T$ is the sampling period.

Speed $v$ is the system output, and the output equation can be written as:

15 $${\varvec{y}}(k) = {\varvec{Cx}}(k)\begin{array}{*{20}c} {} \\ \end{array} {\varvec{C}} = \left( {\begin{array}{*{20}c} 1 & 0 \\ \end{array} } \right)$$

#### Cost and constraint functions

The system control objective is the speed tracking accuracy. At the same time, in order to avoid excessive acceleration, the cost function is defined as:

16 $$\begin{aligned} & J(x(k),u(k - 1),\Delta u(k)) \\ & \quad = \sum\limits_{i = 1}^{{H_{p} }} {\left\| {y_{p} (k + i|k) - y_{ref} (k + i|k)} \right\|}_{Q}^{2} + \sum\limits_{i = 0}^{{H_{c} - 1}} {\left\| {\Delta u(k + i)} \right\|}_{R}^{2} + \sum\limits_{i = 0}^{{H_{c} - 1}} {\left\| {u(k + i)} \right\|}_{S}^{2} \\ \end{aligned}$$

where $k - 1$ is the last sampling time; $H_{p}$ is the prediction step; $H_{c}$ is the control step size; $y_{p} (k + i|k)$ is the predicted value of control output; $y_{ref} (k + i|k)$ is the reference value of control output; $(k + i|k)$ indicates that the value at time $k + i$ is predicted according to the information at time $k$, where $i = 1, \ldots ,H_{p}$; $u(k + i)$ and $\Delta u(k + i)$ are $k + i$ time control input and control input increment respectively, where $i = 0, \ldots ,H_{c} - 1$; $Q,R,S$ are the system matrices of system output, control increment and control weight.

System constraints are mainly acceleration and rate of acceleration change constraints, which can be expressed as:

17 $$u_{\min } \le u(k + i) \le u_{\max } ,i = 0, \ldots ,H_{c} - 1$$

18 $$\Delta u_{\min } \le \Delta u(k + i) \le \Delta u_{\max } ,i = 0, \ldots ,H_{c} - 1$$

where $u_{\min }$ and $u_{\max }$ are acceleration limits, $\Delta u_{\min }$ and $\Delta u_{\max }$ are acceleration limits.

#### MPC optimization model

Under object model Eq. (14), the basic objective of MPC is to minimize the cost function, and time satisfy the control constraints (17) and (18), the cost function (16) is the minimum. Each control cycle solves the following optimization problems:

19 $$\mathop {\min }\limits_{\Delta u(k)} J(x(k),u(k - 1),\Delta u(k))$$

Finally, the optimal input increment $\Delta u$ is obtained, and the optimal input can be expressed as:

20 $$u(k) = u(k - 1) + \Delta u$$

### Positioning algorithm of "INS + Odometer" fusion

Odometer is a device for measuring vehicle speed and distance. It is simple to use, but random noise such as wheel/rail idling and sliding will inevitably occur during the operation of inspection robot, resulting in positioning error. Therefore, a single odometer cannot achieve accurate positioning^34^. Inertial navigation is an autonomous navigation method that does not rely on external information. It can not only provide the position and speed information of the carrier, but also provide the attitude information of the carrier. The data update frequency and the short-term accuracy are high, but the navigation error will increase with time, especially the position error. Therefore, it is necessary to use external information for assistance to realize integrated navigation and effectively reduce the problem of error accumulation over time^35^. In this paper, Kalman filter is used to integrate odometer and inertial sensing unit to monitor the position and attitude information of inspection robot in real time.

#### INS output model

The inertial sensing unit is fixed on the inspection robot, and the inspection robot coordinate system is set as the carrier coordinate system (b system). The forward direction of the inspection robot is $x_{b}$ axes, the direction perpendicular to the moving direction on the plane of the fuselage is $y_{b}$ axes, and the direction perpendicular to the plane of the fuselage is $z_{b}$ axes. Select the geographic coordinate system (g system) as the navigation coordinate system (n system), take the center of gravity of the inspection robot as the center, and specify $x_{n} ,y_{n} ,z_{n}$ to point to the East, North and sky respectively. Among them, the heading angle of the inspection robot is $\varphi$, the pitch angle is $\theta$ and the roll angle is $\gamma$.

The attitude quaternion differential equation of the inspection robot^36^ is expressed as Eq. (21):

21 $$\begin{aligned} & \dot{Q}(t) = \frac{1}{2}Q(t) \otimes (\omega_{nb}^{b} )_{q} \\ & \omega_{nb}^{b} = \omega_{ib}^{b} - C_{n}^{b} \omega_{in}^{b} = \left[ {\begin{array}{*{20}c} {\omega_{x} } \\ {\omega_{y} } \\ {\omega_{z} } \\ \end{array} } \right] - C_{n}^{b} \left[ {\begin{array}{*{20}c} { - \frac{{v_{y} }}{{R_{M} + h}}} \\ {\omega_{ie} \cos L + \frac{{v_{x} }}{{R_{N} + h}}} \\ {\omega_{ie} \sin L\frac{{v_{x} }}{{R_{N} + h}}\tan L} \\ \end{array} } \right] \\ \end{aligned}$$

where $Q(t)$ is the attitude quaternion describing the attitude of the inspection robot; $$\omega_{ib}^{b} = \left[ {\begin{array}{*{20}c} {\omega_{x} } & {\omega_{y} } & {\omega_{z} } \\ \end{array} } \right]^{T}$$ is the angular velocity of the inspection robot measured by the gyroscope; $\omega_{ie}$ is the rotation speed of the earth; $h$ is the altitude of the inspection robot; $v_{x} ,v_{y} ,v_{z}$ are the East, North and sky velocity components of the inspection robot strapdown inertial navigation in the navigation coordinate system; $L$ is the latitude of the inspection robot on the earth; $(\omega_{nb}^{b} )_{q}$ is expressed as the quaternion form of vector; $R_{M}$ and $R_{N}$ are the radius of curvature of the earth's meridian circle and Mao unitary circle where the inspection robot is located.

According to the attitude conversion matrix of inspection robot:

22 $$\begin{aligned} C_{b}^{n} & = \left[ {\begin{array}{*{20}c} {\cos \gamma \cos \varphi + \sin \gamma \sin \varphi \sin \theta } & {\sin \varphi \cos \theta } & {\sin \gamma \cos \varphi - \cos \gamma \sin \varphi \sin \theta } \\ { - \cos \gamma \sin \varphi + \sin \gamma \cos \varphi \sin \theta } & {\cos \varphi \cos \theta } & {\sin \gamma \sin \varphi - \cos \gamma \cos \varphi \sin \theta } \\ { - \sin \gamma \cos \theta } & {\sin \theta } & {\cos \gamma \cos \theta } \\ \end{array} } \right] \\ & = \left[ {\begin{array}{*{20}c} {T_{11} } & {T_{12} } & {T_{13} } \\ {T_{21} } & {T_{22} } & {T_{23} } \\ {T_{31} } & {T_{32} } & {T_{33} } \\ \end{array} } \right] \\ \end{aligned}$$

The obtained attitude angle is:

23 $$\begin{aligned} \theta & = \sin^{ - 1} (T_{32} ) \\ \gamma & = - \tan (T_{31} /T_{33} ) \\ \varphi & = \tan^{ - 1} (T_{12} /T_{22} ) \\ \end{aligned}$$

The differential equation of speed update of inspection robot is:

24 $$\begin{aligned} \dot{v}_{x} & = f_{x} + \left( {2\omega_{ie} \sin L + \frac{{v_{x} }}{{R_{N} + h}}\tan L} \right)v_{y} - \left( {2\omega_{ie} \cos L + \frac{{v_{x} }}{{R_{N} + h}}} \right)v_{z} \\ \dot{v}_{y} & = f_{y} - \left( {2\omega_{ie} \sin L + \frac{{v_{x} }}{{R_{N} + h}}\tan L} \right)v_{x} - \frac{{v_{x} }}{{R_{N} + h}}v_{z} \\ \dot{v}_{z} & = f_{z} + \left( {2\omega_{ie} \cos L + \frac{{v_{x} }}{{R_{N} + h}}} \right)v_{x} + \frac{{v_{y}^{2} }}{{R_{M} + h}} - g \\ \end{aligned}$$

where $$f = \left[ {\begin{array}{*{20}c} {f_{x} } & {f_{y} } & {f_{z} } \\ \end{array} } \right]^{T}$$ is the specific force measured by accelerometer; $g$ is the gravitational acceleration of the position of the inspection robot.

The position update differential equation of inspection robot is:

25 $$\begin{aligned} \dot{L} & = {{v_{y} } \mathord{\left/ {\vphantom {{v_{y} } {(R_{M} + h)}}} \right. \kern-0pt} {(R_{M} + h)}} \\ \dot{\lambda } & = {{v_{x} } \mathord{\left/ {\vphantom {{v_{x} } {[(R_{N} + h)cosL]}}} \right. \kern-0pt} {[(R_{N} + h)cosL]}} \\ \dot{h} & = v_{z} \\ \end{aligned}$$

Through the above method, the attitude, velocity and position equations after inertial navigation can be obtained. Next, the odometer output model is analyzed.

#### Odometer output model

The odometer usually outputs the mileage increment within the sampling time interval in pulse mode, so that the position increment output direction of the odometer always points to the running direction of the inspection robot. The coordinate system of the odometer on the inspection robot is set as the m system, which is the right front up coordinate system fixedly connected with the inspection robot, that is, the $ox$ axis is forward along the longitudinal axis of the inspection robot, the $oy$ axis is right along the transverse axis of the inspection robot, and the $oz$ axis is upward along the vertical ground. The number of pulses output by the odometer in the increment of position in the m system projection vector is:

26 $$N^{m} = \left[ {\begin{array}{*{20}c} 0 & {\frac{2\pi Rn(t)}{N}} & 0 \\ \end{array} } \right]^{T}$$

where $N$ is the number of pulses generated by one rotation of the odometer. $n(t)$ is the number of pulses generated per unit time t, and the radius of the indexing circle of the wheelset is R.

The inertial navigation coordinate system is b system. The rotation matrix of converting the odometer coordinate system into the inertial navigation coordinate system is $C_{m}^{b}$. If $K_{D}$ is the odometer scale factor, the projection of the odometer output position increment in the b system is:

27 $$\Delta S_{i}^{b} = C_{b}^{m} K_{D} N^{m}$$

After further sorting, the position increment of odometer is calculated as follows:

28 $$\Delta S_{i}^{b} = \left[ {\begin{array}{*{20}c} {\Delta S_{i}^{bx} } \\ {\Delta S_{i}^{by} } \\ {\Delta S_{i}^{bz} } \\ \end{array} } \right] = \left[ {\begin{array}{*{20}c} {\sin \alpha_{\varphi } \cos \alpha_{\theta } } \\ {\cos \alpha_{\varphi } \cos \alpha_{\theta } } \\ {\sin \alpha_{\theta } } \\ \end{array} } \right]K_{D} \frac{2\pi Rn(t)}{N}$$

wherein $\alpha_{\varphi }$ and $\alpha_{\theta }$ are the heading and pitch installation angle of the inspection robot coordinate system relative to the odometer coordinate system.

Considering only scale factor error $\delta K_{D}$ and installation error angle $\delta \alpha_{\varphi }$ and $\delta \alpha_{\theta }$. These errors are considered to be small. The components under the coordinate system of the inspection robot actually output by the odometer can be simplified as follows:

29 $$\Delta \tilde{S}_{i}^{b} = \left[ {\begin{array}{*{20}c} 1 & {\delta \alpha_{\varphi } } & 0 \\ { - \delta \alpha_{\varphi } } & 1 & {\delta \alpha_{\theta } } \\ 0 & { - \delta \alpha_{\theta } } & 1 \\ \end{array} } \right](1 + \delta K_{D} )(\Delta S_{i}^{b} + w_{d} )$$

where $w_{d}$ is random noise interference.

The second-order small quantity can be ignored. The component under the inspection robot coordinate system of the actual output of the odometer can be expressed as:

30 $$\begin{aligned} & & \Delta \tilde{S}_{i}^{b} = \Delta S_{i}^{b} + M_{i}^{b} \left[ {\begin{array}{*{20}c} {\delta K_{D} } \\ {\delta \alpha_{\varphi } } \\ {\delta \alpha_{\theta } } \\ \end{array} } \right] + w_{d} \\ & M_{i}^{b} = \left[ {\begin{array}{*{20}c} {\Delta S_{i}^{bx} } & 0 & {\Delta S_{i}^{by} } \\ {\Delta S_{i}^{by} } & {\Delta S_{i}^{bz} } & { - \Delta S_{i}^{bx} } \\ {\Delta S_{i}^{bz} } & { - \Delta S_{i}^{by} } & 0 \\ \end{array} } \right] \\ \end{aligned}$$

#### Kalman system model

This paper establishes the system model under the position observation mode. The errors included in the combined system are mainly the attitude, speed and position errors of inertial navigation, the zero-drift error of gyroscope and angular velocimeter, the odometer scale factor error and the installation error of pitch angle and heading angle. The established combined positioning system is interrelated with these errors, forming a closed-loop feedback system to optimize and correct the errors and improve the positioning accuracy.

The equation of Kalman filter system is established as follows^37^:

31 $$\dot{X}(t) = F(t)X(t) + G(t)w(t)$$

where $F(t)$ is the system matrix; $w(t)$ is system noise, $G(t)$ is the system noise transfer matrix, which is generally white noise.

The integrated navigation state variable is 18 dimensions:

32 $$X = \left[ {(\delta v^{n} )^{T} ,(\varphi^{n} )^{T} ,(\delta p)^{T} ,(\varepsilon^{b} )^{T} ,(\nabla^{b} )^{T} ,\delta K_{D} ,\delta \alpha_{\theta } ,\delta \alpha_{\varphi } } \right]^{T}$$

where $v^{n}$ is the speed error; $\varphi^{n}$ is attitude error; $\delta p$ is the position error; $\varepsilon^{b}$ is gyro zero drift; $\nabla^{b}$ is the zero drift of accelerometer; $\delta K_{D}$ is the odometer scale factor error; $\delta \alpha_{\theta } ,\delta \alpha_{\varphi }$ is the installation error angle of pitch and heading.

The odometer scale factor error and installation error angle can be regarded as random constants. Therefore, the corresponding system matrix is:

33 $$F = \left[ {\begin{array}{*{20}c} {F_{1} } & {0_{3 \times 3} } \\ {0_{3 \times 15} } & {0_{3 \times 3} } \\ \end{array} } \right]$$

$F_{1}$ is the transfer matrix corresponding to the inertial navigation system error equation, and the calculation method is shown in reference^37^.

Inertial navigation position update needs to calculate the position increment in the navigation coordinate system of each cycle, and the formula for calculating the position increment is:

34 $$\Delta P_{i}^{s} = (V_{i}^{b} + v^{n} )T$$

where $V_{i}^{b}$ and $V_{i - 1}^{b}$ are the coordinate system speeds of the inspection robot obtained from the pure inertial navigation solution at $t_{i}$ and $t_{i - 1}$ times respectively; $T$ is the update cycle.

The updating algorithm formula of dead reckoning position output by odometer is:

35 $$\begin{aligned} \Delta P_{i}^{D} & = C_{b}^{n} (t_{i - 1} )\Delta \tilde{S}_{i}^{b} + C_{b}^{n} (t_{i - 1} )\Delta S_{i}^{b} \varphi^{n} \\ & = C_{b}^{n} (t_{i - 1} )\Delta S_{i}^{b} + C_{b}^{n} (t_{i - 1} )\Delta S_{i}^{b} \varphi^{n} + C_{b}^{n} (t_{i - 1} )M_{i}^{b} \left[ {\begin{array}{*{20}c} {\delta K_{D} } \\ {\delta \alpha_{\varphi } } \\ {\delta \alpha_{\theta } } \\ \end{array} } \right] + C_{b}^{n} (t_{i - 1} )w_{d} \\ \end{aligned}$$

where $C_{b}^{n} (t_{i - 1} )$ is the attitude matrix solved by the inertial navigation system at time $t_{i - 1}$.

The difference between ins and dead reckoning position increment in unit time is used as the measurement to expand the dead reckoning error to the state. Since the differential calculation is used for the inertial navigation position calculation, and the error accumulation increases with the increase of time, the difference per second between the inertial navigation position increment and the dead reckoning position increment under the navigation coordinate system is used as the measured value:

36 $$Z = \sum\limits_{i = 1}^{K} {\Delta P_{i}^{S} - \sum\limits_{i = 1}^{K} {\Delta P_{i}^{D} } }$$

where $K$ is the number of location updates in T.

During the operation of the inspection robot, the real position of the inertial navigation solution is the same as that of the odometer dead reckoning: $V_{i}^{T} T = \sum\nolimits_{i = 1}^{K} {(C_{b}^{n} (t_{i - 1} )\Delta S_{i}^{b} )}$. According to the formula (35), and considering that T time is very short, the inertial navigation error states $v^{n}$, $\varphi^{n}$, $\delta \alpha_{\varphi }$ and $\delta \alpha_{\theta }$ can be approximated as constants, the measurement equation of navigation is further summarized as:

37 $$\begin{gathered} Z = HX + V \\ H(t) = \left[ {\begin{array}{*{20}c} {KT \times I_{3 \times 3} } \\ { - \sum\limits_{i = 1}^{K} {(C_{b}^{n} (t_{i - 1} )\Delta S_{i}^{b} ) \times 0_{3 \times 6} } } \\ { - \sum\limits_{i = 1}^{K} {(C_{b}^{n} (t_{i - 1} )M_{i}^{b} )} } \\ \end{array} } \right] \\ V = - \sum\limits_{i = 1}^{K} {C_{b}^{n} (t_{i - 1} )w_{d} } \\ \end{gathered}$$

According to the above state equation and measurement equation, the Kalman filter algorithm is used to continuously modify the position and attitude of the inspection robot, so as to realize the accurate positioning of the inspection robot in the tunnel.

## Verification and application

**Figure 5:**
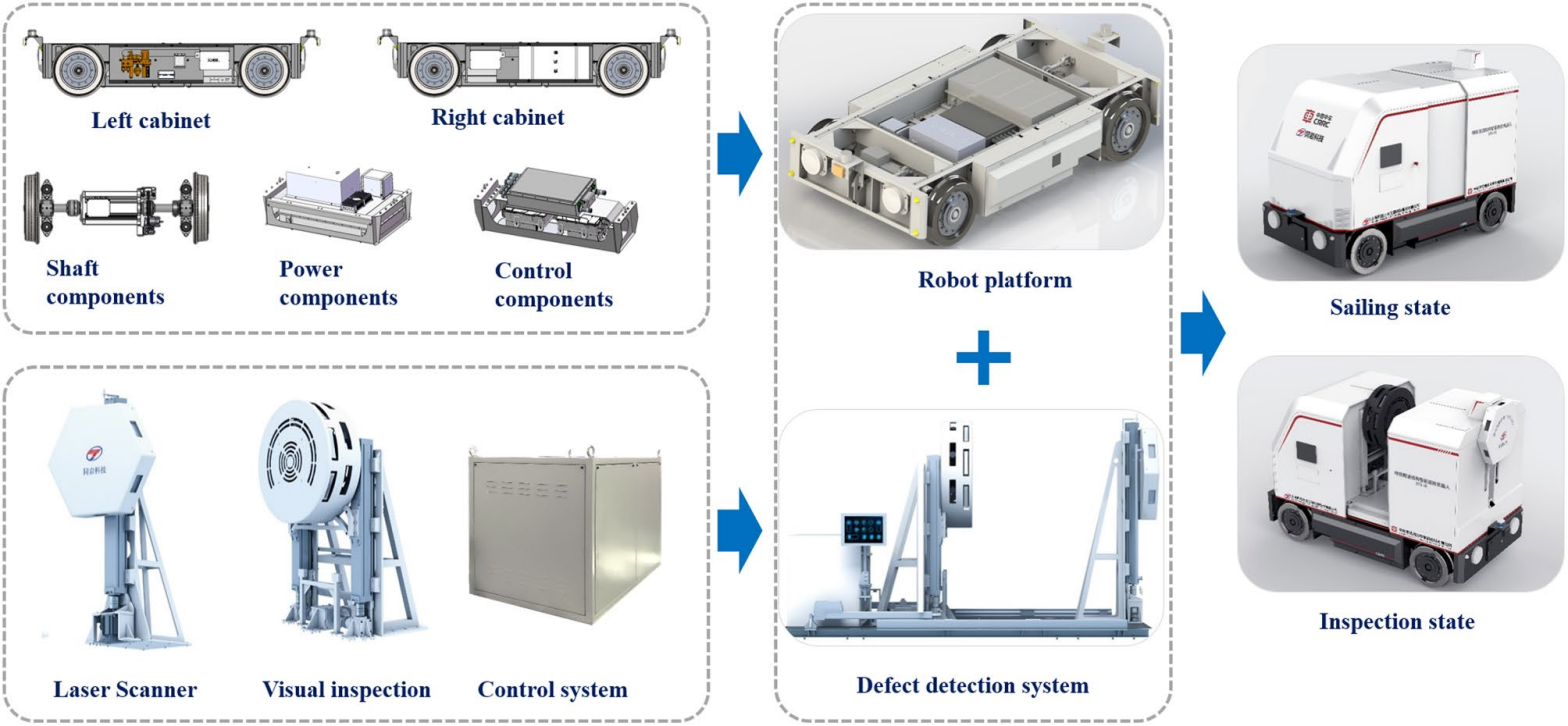
Assembling of tunnel inspection robot.

**Figure 6:**
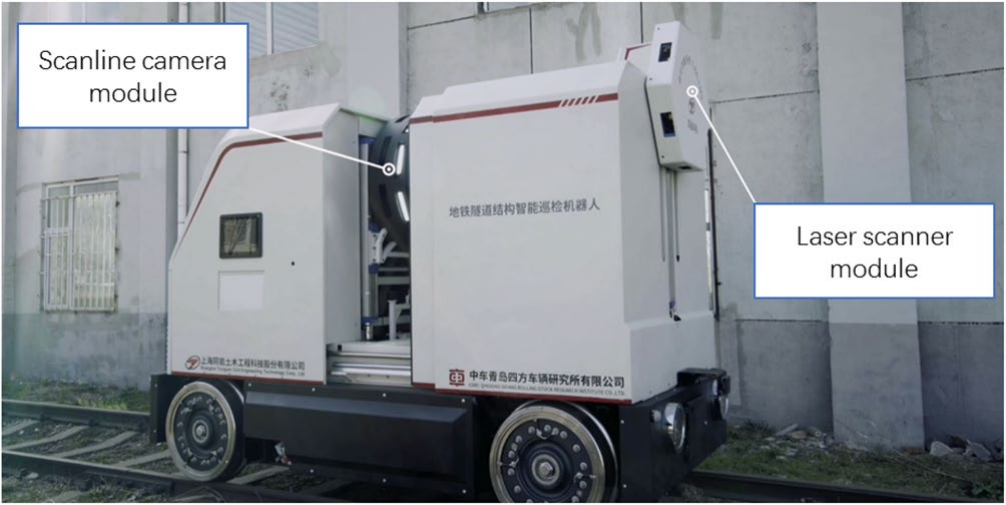
Tunnel inspection robot.

As shown in Fig. 5, the tunnel inspection robot has two modes, i.e. sailing and inspection. The real tunnel inspection robot used in this test is shown in Fig. 6. It is equipped with driverless controller, 4G module, motor controller, brake, inertial sensor and odometer. The experimental site is Dalian factory. It mainly carries out two aspects of testing, one is to verify the speed planning and speed following strategy, and the other is to verify the accuracy of the fusion positioning of inertial sensor and odometer. The scanline camera module can acquire tunnel structure surface image and identify structure defects. The laser scanner module acquire tunnel outline information and detect deformation and invasion.

The speed planning and tracking algorithms of the tunnel inspection robot as well as the multi-sensor fusion localization algorithms are tested on the actual track line.

*Case 1*: In order to verify the effect of speed planning and speed tracking algorithm, the test was carried out on the 1.5 km railway test line. Firstly, the speed planning algorithm is used to plan the motion process of acceleration, uniform speed and braking. The displacement target is set to be 1 km and the limit speed is 60 km/h. The two-wa Doppler radar (speed measurement range 0–120 km/h, accuracy 0.05%) is used to monitor the running speed of the robot. The time, speed, expected acceleration, jerk and other physical quantities during the test are recorded The PID algorithm and MPC algorithm are respectively used for speed tracking control. Repeat 50 times.

**Figure 7:**
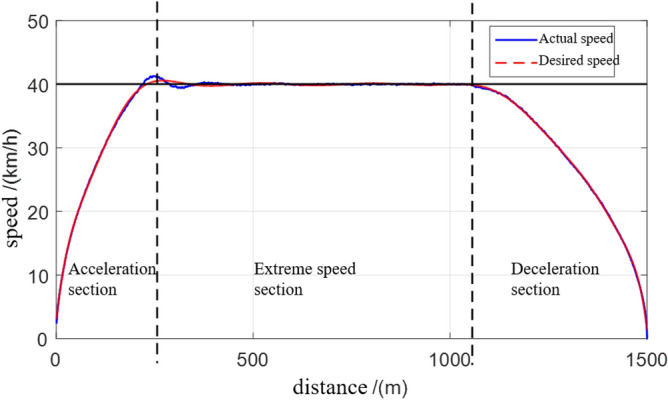
Velocity variation curve.

**Figure 8:**
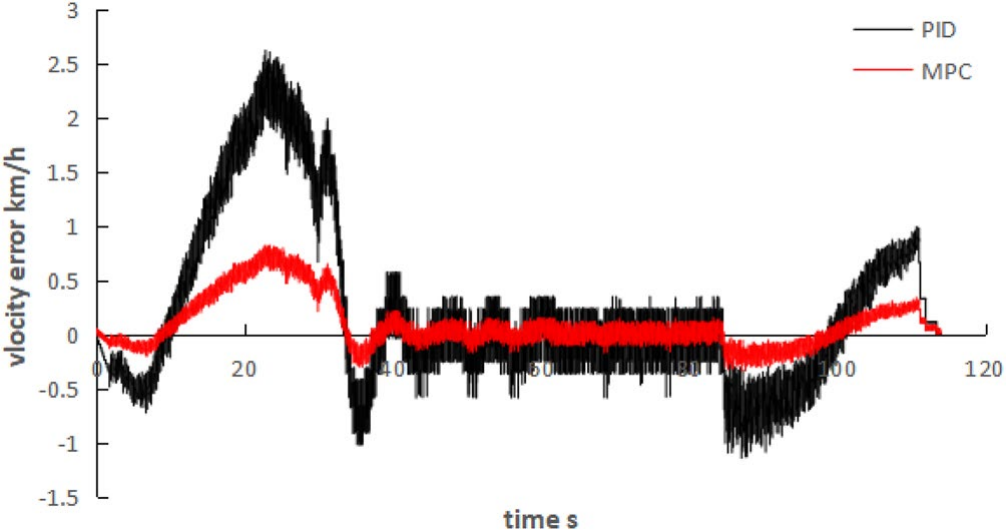
Velocity Error between PID and MPC.

It can be seen from Table 1, Figs. 7 and 8, the overshoot error of the proposed algorithm is 0.89%, the stability error is 0.32%, and the change amount of the control quantity is reduced at the same time. It is verified that compared with the traditional PID algorithm, the MPC speed tracking algorithm can reduce the overshoot, the control error and the change rate of the control output, thus improving the speed following accuracy and stability.

**Table 1:** Results analysis table of PID and the speed tracking algorithm in this paper (average of 50 tests).

Comparison of items	PID	MPC
Error of overshoot (%)	4.2%	0.89%
Steady state error (%)	0.8%	0.32%
Jerk (m/s^3^)	0.6	0.2

*Case 2*: In order to verify the advantages of the positioning fusion algorithm, the experiment was carried out on a 2 km railway test loop. The running speed of the vehicle is 40 km/h, and the actual running distance is based on the 100-m scale (According to the railway construction specifications and the field survey confirmation, the positioning accuracy of 100-m scale is within 0.01 m). When each 100-m mark was reached, the distance recorded by the odometer and the distance calculated by the algorithm in this paper were respectively recorded. The test was repeated 50 times, and the maximum error and average error were compared.

As can be seen from Table 2, the proposed algorithm has a maximum positioning error of 0.15%, an average error of 0.08%, and a mean square error of 1.7 m within 2 km, which verifies that the multi-sensor fusion positioning algorithm has significantly improved its accuracy compared with the single odometer positioning algorithm, and cannot effectively make up for the position errors caused by creep and sensor errors.

**Table 2:** Analysis table of positioning test results.

Comparison of items	Single mileage calculation	Multi-sensor fusion algorithm
Maximum error of 50 tests (%)	4.213%	0.153%
Average error of 50 tests (%)	3.522%	0.079%
Mean square error of 50 tests (m)	10.647	1.677

**Figure 9:**
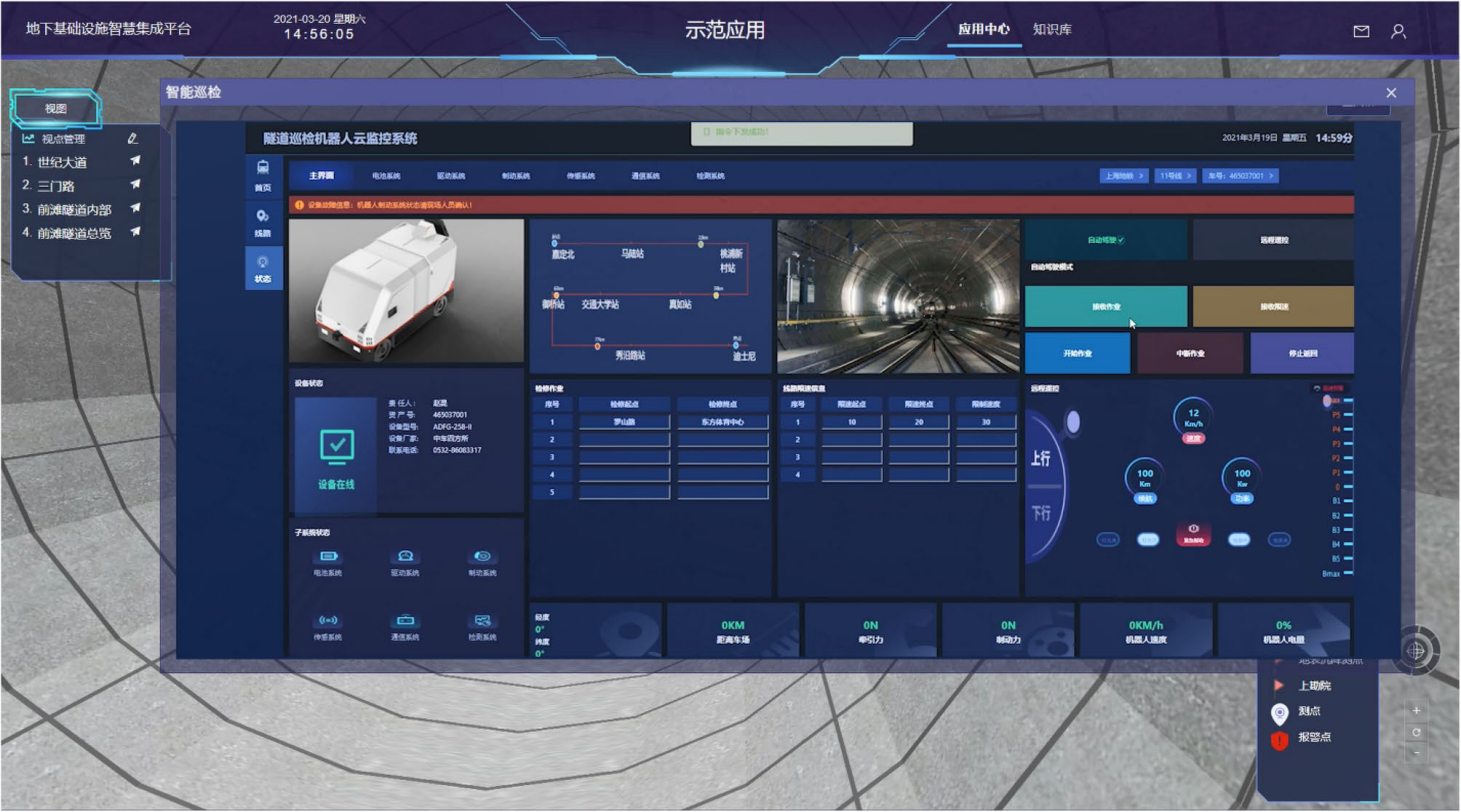
Application of tunnel inspection robot with the cloud platform.

The integrated parameter optimization approach was integrated into the metro tunnel structure defect inspection platform based on iS3^38^, as shown in Fig. 9. With the help of the cloud platform, the robot can automatically sail and detect in the tunnel between two stations. On the other hand, the data acquired from the robot simultaneously transfer to backstage analysis system and a just-in-time maintenance service will be online.

## Conclusions

To sum up, the motion control and positioning system of metro tunnel inspection robot can realize the remote control and stable speed control of the robot in the metro tunnel environment through trapezoidal speed planning algorithm and MPC based speed tracking algorithm, and realize the autonomous positioning of the inspection robot in the tunnel environment through the positioning algorithm integrating inertial sensor and odometer without adding external road signs. In the test, the stable error of velocity accuracy reaches 0.32% and the average error of positioning accuracy reaches 0.08% which solves the localization and speed tracking problems of the tunnel inspection robot in the complex tunnel environment. Although the positioning accuracy and stability of the combination of inertial navigation and odometer have been improved, equipment errors still exist and will accumulate during long-term operation. The next step is to consider using machine vision to identify tunnel features for positioning correction to reduce the accumulated errors.

## Data Availability

The data that support the findings of this study are available from the corresponding author upon reasonable request.
